# Kv2.1 Channels Prevent Vasomotion and Safeguard Myogenic Reactivity in Rat Small Superior Cerebellar Arteries

**DOI:** 10.3390/cells12151989

**Published:** 2023-08-02

**Authors:** Kristina Här, Natalia N. Lysenko, Daniela Dimitrova, Torsten Schlüter, Olga Zavaritskaya, Andrej G. Kamkin, Mitko Mladenov, Olaf Grisk, Ralf Köhler, Hristo Gagov, Rudolf Schubert

**Affiliations:** 1 European Center of Angioscience (ECAS), Research Division Cardiovascular Physiology, Medical Faculty Mannheim, Heidelberg University, 68167 Mannheim, Germany; 2 Department of Physiology, N. I. Pirogov Russian National Research Medical University, 117997 Moscow, Russia; 3 Institute of Biophysics and Biomedical Engineering, Bulgarian Academy of Sciences, 1113 Sofia, Bulgaria; 4 Institute of Physiology, Universitätsmedizin Greifswald, 17475 Greifswald, Germany; 5 Institute of Biology, Faculty of Natural Sciences and Mathematics, University of Ss. Cyril and Methodius, 1000 Skopje, North Macedonia; 6 Institute of Physiology, Brandenburg Medical School Theodor Fontane, 16816 Neuruppin, Germany; 7 ARAID-IACS, UIT University Hospital Miguel Servet, 50009 Zaragoza, Spain; 8 Department of Animal and Human Physiology, Faculty of Biology, Sofia University ‘St. Kliment Ohridski’, 1164 Sofia, Bulgaria; 9 Physiology, Institute of Theoretical Medicine, Faculty of Medicine, University of Augsburg, Universitätsstrasse 2, 86159 Augsburg, Germany

**Keywords:** small arteries, smooth muscle cells, myogenic response, voltage-gated potassium channels

## Abstract

Vascular smooth muscle voltage-gated potassium (Kv) channels have been proposed to contribute to myogenic autoregulation. Surprisingly, in initial experiments, we observed that the Kv2 channel inhibitor stromatoxin induced vasomotion without affecting myogenic tone. Thus, we tested the hypothesis that Kv2 channels contribute to myogenic autoregulation by fine-tuning the myogenic response. Expression of Kv2 channel mRNA was determined using real-time PCR and ‘multiplex’ single-cell RT-PCR. Potassium currents were measured using the patch-clamp technique. Contractile responses of intact arteries were studied using isobaric myography. Expression of Kv2.1 but not Kv2.2 channels was detected in intact rat superior cerebellar arteries and in single smooth muscle cells. Stromatoxin, a high-affinity inhibitor of Kv2 channels, reduced smooth muscle Kv currents by 61% at saturating concentrations (EC_50_ 36 nmol/L). Further, stromatoxin (10–100 nmol/L) induced pronounced vasomotion in 48% of the vessels studied. In vessels not exhibiting vasomotion, stromatoxin did not affect myogenic reactivity. Notably, in vessels exhibiting stromatoxin-induced vasomotion, pressure increases evoked two effects: First, they facilitated the occurrence of random vasodilations and/or vasoconstrictions, disturbing the myogenic response (24% of the vessels). Second, they modified the vasomotion by decreasing its amplitude and increasing its frequency, thereby destabilizing myogenic tone (76% of the vessels). Our study demonstrates that (i) Kv2.1 channels are the predominantly expressed Kv channels in smooth muscle cells of rat superior cerebellar arteries, and (ii) Kv2.1 channels provide a novel type of negative feedback mechanism in myogenic autoregulation by preventing vasomotion and thereby safeguarding the myogenic response.

## 1. Introduction

In many organs, blood flow and capillary hydrostatic pressure do not change very much during alterations in perfusion pressure. This mechanism allows the blood perfusion of an organ to be matched with its metabolic demand independently of variations in systemic arterial pressure. A significant part of this effect is governed by the myogenic response of small arteries and arterioles. Moreover, alterations of the myogenic response are associated with several widespread pathological conditions, like chronic heart failure, diabetes, arterial hypertension, and stroke (for reviews, see, e.g., [1,2,3,4,5]). Thus, the myogenic response has an important functional impact on the cardiovascular system under physiological as well as pathophysiological conditions.

The myogenic response is mediated by the delicately orchestrated interaction of two main pathways. Firstly, a Ca^2+^-dependent pathway including membrane potential depolarization and a subsequent elevation of intracellular Ca^2+^ mainly based on Ca^2+^-influx. Secondly, several signaling pathways that regulate the Ca^2+^ sensitivity of the contractile apparatus, e.g., PKC, RhoA/Rho kinase/ROS, finally led to a rise in MLC phosphorylation and subsequent vessel constriction [2,3,4,5,6,7,8]. Interestingly, vascular smooth muscle, large conductance calcium-activated potassium (BK) channels, and voltage-gated potassium (Kv) channels have been suggested to modulate the myogenic response. They serve as a negative feedback mechanism, limiting myogenic vasoconstriction. Thus, they prevent excessive vasoconstriction or vasospasm (e.g., [9,10,11,12,13,14,15,16,17,18,19,20,21,22,23,24,25]).

Kv channels are protein complexes formed by four pore-forming α-subunits and accessory subunits, including, for example, β-subunits, KCHIP, and minK. The family of Kv channels consists of several subfamilies (Kv1-Kv12) [26]. In small arteries of the systemic circulation, the existence of a number of Kv subunits has been observed on the mRNA and protein levels (see, for example, reviews [27,28,29,30]). However, knowledge about the functional role of particular vascular smooth muscle Kv channel subfamilies is still fragmentary, mostly due to the lack of subunit-specific inhibitors.

In a series of studies using different Kv1 channel inhibitors and transfection of dominant-negative Kv1.5 mutants, Kv1 channels have been reported to weaken the myogenic response as a consequence of a negative feedback mechanism at which pressure-induced depolarization activates Kv1 channels [15,19,22]. Recent studies using the high-affinity Kv2 channel inhibitor stromatoxin [31] suggested that Kv2 channels also have an impact on myogenic tone in rat middle cerebral arteries [11] and mouse posterior cerebral arteries [32]. Moreover, using rat middle cerebral arteries, it was shown that heteromeric complexes of Kv2.1/Kv9.3 channels weaken myogenic tone between 10 and 100 mmHg. This pressure range is larger than the one where Kv1 channels weaken myogenic tone [24,25]. Moreover, Kv7 channels have also been shown to blunt myogenic tone in, for example, rat middle cerebral arteries [14,25] and rat gracilis arteries [20]. In summary, the data available so far propose that vascular smooth muscle Kv1, Kv2, and Kv7 channels activated by pressure-induced depolarization and thereby producing re-polarization constitute negative feedback that weakens myogenic autoregulation and thus counteracts excessive vasoconstriction or vasospasm.

Surprisingly, in preliminary experiments, we observed that the Kv2 channel inhibitor stromatoxin did not produce the expected increase in myogenic tone but destabilized myogenic tone by inducing vasomotion in rat superior cerebellar arteries. Therefore, we tested the hypothesis that Kv2 channels contribute to myogenic autoregulation by fine-tuning the myogenic response (the Bayliss effect).

## 2. Materials and Methods

### 2.1. Dissection and Mounting of Vessels

The investigation conforms to the *US Guide for the Care and Use of Laboratory Animals* (Eighth Edition, National Academy of Sciences, 2011). Approval for the use of laboratory animals in this study was granted by a governmental committee on animal welfare (I-17/17). Male Wistar rats (10–14 weeks; Charles River Laboratories, Sulzfeld, Germany) were killed under CO_2_ narcosis. Small superior cerebellar arteries were isolated and mounted in an experimental chamber containing an experimental solution (PSS) consisting of (in mmol/L): 146 NaCl, 4.5 KCl, 1.2 NaH_2_PO_4_, 1.0 MgSO_4_, 1.6 CaCl_2_, 0.025 EDTA, 5.5 glucose, and 5 HEPES at pH 7.4. The microscope image of the vessel was viewed with a CCD camera, digitized by a frame-grabber board, and diameter reactions were analyzed online as described previously [33].

### 2.2. Experimental Protocol

The vessels were pressurized to 80 mmHg. Leaking vessels were discarded at any stage of the experiment to ensure complete non-flow conditions. The temperature was set to 37.0 ± 0.5 °C, and the pH was set to 7.40 ± 0.05. After spontaneous myogenic tone had developed (constriction to a diameter between 50 and 80% of the fully relaxed diameter at 80 mmHg in Ca^2+^-free PSS; the fully relaxed diameter was in the range of 170–300 µm), vessel viability was tested obligatorily in each vessel with 1 µmol/L bradykinin (to test functionality of the endothelium, see below) and 0.1 µmol/L serotonin (viability criterion: at least 25% constriction). In most experiments, the endothelium was removed by passing an air bubble through the vessel lumen (successful functional removal of the endothelium was shown by the absence of a relaxation to 1 µmol/L bradykinin), and the influence of sensory nerve endings was eliminated by pretreatment with 10 µmol/L capsaicin for 30 min. Thereafter, the desired experiments were performed. Data analysis was performed by calculating (i) normalized diameter = (d_act_/d_pas_(80)), where d_act_ and d_pas_(80) are the vessel diameters of the myogenically active vessel at the desired pressure in PSS and the passive vessel at 80 mmHg in Ca^2+^-free solution, respectively, and (ii) myogenic tone = (1 − d_act_/d_pas_), where d_act_ and d_pas_ are the vessel diameters of the myogenically active and passive vessel at the same pressure.

### 2.3. Cell Isolation

A piece of a small superior cerebellar artery was placed into a microtube containing 1 mL of an isolation solution containing (in mmol/L): 55 NaCl, 6 KCl, 88 Na glutamate, 2 MgCl_2_, 10 HEPES, 10 glucose, pH 7.4, as well as 0.6 mg/mL papain and 1.2 mg/mL DL-dithiothreitol for 20 min at 37 °C. Then, the artery was transferred into a microtube containing 1 mL of the isolation solution supplemented with 1.2 mg/mL collagenase F, 1.0 mg/mL trypsin inhibitor, and 0.5 mg/mL elastase for 12 min at 37 °C. Single cells were released by trituration with a polyethylene pipette into the experimental bath solution consisting of (in mmol/L): 126 NaCl, 4.5 KCl, 1 MgCl_2_, 0.1 CaCl_2_, 10 HEPES, 20 taurine, 20 glucose, and 5 pyruvate at pH 7.4. The pipette solution contained (in mmol/L): 109 KCl, 10 NaCl, 1 MgCl_2_, 1 CaCl_2_, 10 EGTA (purity 96%), and 10 HEPES, giving a free calcium concentration < 10^−7^ mol/L at pH 7.4.

### 2.4. Patch-Clamp Recording

All experiments were performed at room temperature in whole-cell mode. Patch pipettes had resistances of 2–5 MΩ. The recordings were made with an Axopatch 200 amplifier (Axon Instruments, Burlingame, CA, USA). Stimulation of currents and data analysis were conducted with the software package ISO2. To reduce the contribution of BK channels, 10^−7^ mol/L iberiotoxin, a specific inhibitor of these channels, was added to the bath solution [34]. Initial experiments had shown that neither 1 µmol/L glibenclamide, an inhibitor of ATP-sensitive potassium channels, nor 10 µmol/L barium, an inhibitor of inward rectifying potassium channels, produced any effect on the outward current of these cells.

### 2.5. Reverse Transcription and Whole Vessel Real-Time-PCR

RNA was isolated from whole vessel segments of small superior cerebellar arteries using the RNAqueousTM-4PCR kit (Ambion, Austin, TX, USA) according to the manufacturer’s instructions. Reverse transcription was performed with the High-Capacity cDNA Archive Kit (Applied Biosystems, Foster City, CA, USA). RNA samples were reverse transcribed with random hexamer primers in a total volume of 100 µL according to the manufacturer’s instructions. The following primers and probes were designed to span an intron to avoid amplification of genomic DNA: Kv2.1 (GenBank™ accession: NM_013186) 5′primer: CAGATGAACGAGGAGCTGAAGC, 3′primer: TCTGGCCGAACTCGTCTAGG, probe: TCTGGGATCTGCTGGAGAAGCCCA; Kv2.2 (GenBank™ accession: NM_054000) 5′primer: CTGCCAAGCCAGATACCATCAG, 3′primer: GTTGCCCAAATTCATCGTTTTCT, probe: TCGTGTCTATCCTGTTCATCGTCCTTTCCA; porphobilinogen deaminase (Pbgd; GenBank™ accession: X06827) 5′primer: TGGGCACCCGGAAGAGT; 3′primer: CCTGTGGTGGACATAGCAATGAT; probe: CAGACCGACACTGTGGTAGCGATGCT.

Probes were labeled with FAM as the reporter and TAMRA as the quencher. In preliminary experiments, concentrations of primers and probes were optimized to give low threshold values (reporter fluorescence significantly above baseline fluorescence) and high PCR efficiency. Real-time PCR was performed with the ready-to-use TaqMan Universal PCR Master Mix (Applied Biosystems, Foster City, CA, USA) using 2.5 µL of cDNA as a template and the GenAmp 5700 Sequence Detection System (Applied Biosystems, Foster City, CA, USA). The PCR included a 10 min period for activation of the DNA polymerase at 95 °C, 40 cycles at 95 °C for 15 s (denaturation), and 1 min at 60 °C (annealing and extension). Each PCR was performed in triplicate. Non-template controls were included in each PCR run, and the correct amplification of genes was confirmed by standard agarose gel electrophoresis. Data analysis was carried out as described previously [35], using PDGB expression values as an endogenous reference for normalization.

### 2.6. Reverse Transcription and Single-Cell RT-PCR

Harvesting of freshly dispersed smooth muscle cells from small superior cerebellar arteries, reverse transcription of mRNA, and two-round “multiplex” single-cell RT-PCR were performed as described previously [36]. Primer pairs for myosin heavy chain (rMyHC) as a smooth muscle cell marker were stated elsewhere [36]. Since expected PCR fragments for a larger splice variant of rMyHC (160 bp) and rKv2.2 (163 bp) but not for rKv2.1 (207 bp) are of similar length, an additional second-round PCR was performed using primers for rKv2.2. The first and ‘nested’ primer pairs for Kv2.1 and Kv2.2 span intronic sequences. Primer:

rKv2.1:F5′-CAGCCAGGAGCTGGACTACT-3′;R5′-GAGGACAGGAACCTCAGCAA-3′; nested:F5′-AAGGAGCAGATGAACGAGGA-3′;R5′-GTTGAGTGACAGAGCGATGG-3′; (GenBank™ accession: NM_013186);

rKv2.2:F5′-GCCAAGCCAGATACCATCAG-3′;R5′-AGCTCCGGAAGTGTGTTGAG-3′; nested:F5′-TAGAAGGGAGGCAGAGACCA-3′;R5′-GGAAAGGACGATGAACAGGA-3′. (GenBank™ accession: M77482).

### 2.7. Drugs and Chemicals

Serotonin, bradykinin, albumin, collagenase, trypsin inhibitor, DL-dithiothreitol, and the salts for the solutions were obtained from Sigma. Papain was purchased from FERAK (Berlin, Germany). Elastase was from Serva, and iberiotoxin and stromatoxin were from Alomone Labs.

### 2.8. Statistics

All data are means ± SE. One vessel was taken from each rat; thus, n is the number of rats or the number of cells, respectively. Statistical analysis was performed using repeated measures ANOVA and unpaired *t*-tests as appropriate (SPSS 15.0 for Windows).

## 3. Results

### 3.1. Kv2 Channel Expression in Smooth Muscle Cells of Small Superior Cerebellar Arteries

**Figure 1:**
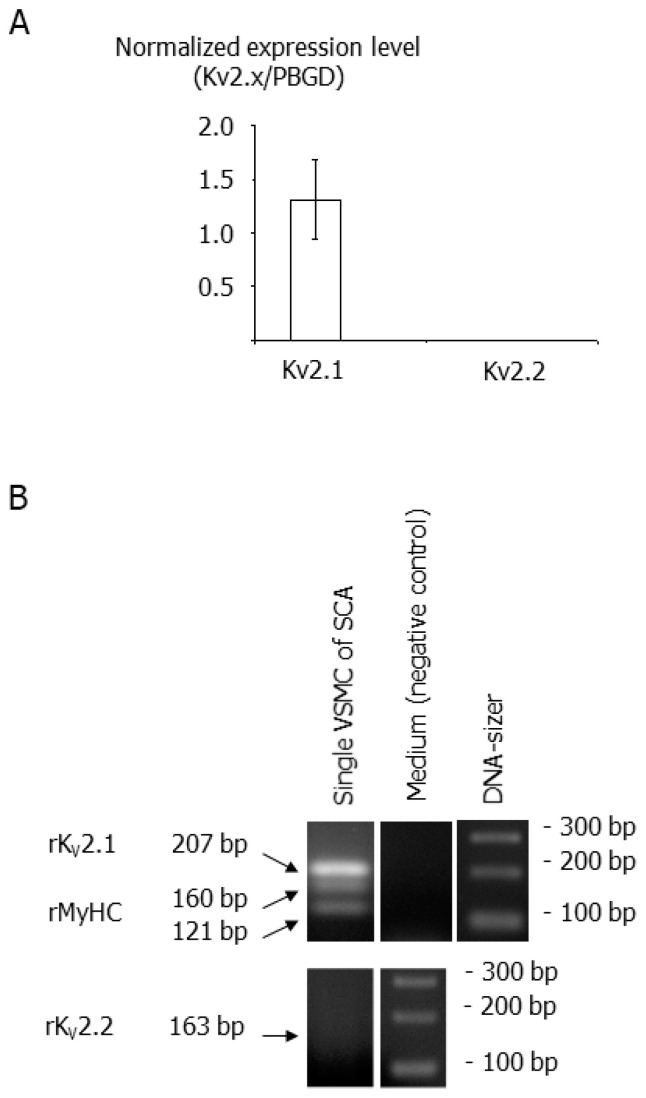
Kv2 channel expression in smooth muscle cells of small superior cerebellar arteries. (**A**) Real-time PCR of Kv2.1 (*n* = 8) and Kv2.2 (*n* = 10) channels in whole vessel segments. PBDG served as an endogenous reference for normalization. (**B**) ‘Multiplex’ single-cell RT-PCR of Kv2.1 (10 out of 11 cells) and Kv2.2 (0 out of 11 cells) on single smooth muscle cells. Smooth muscle cells were identified by positive rMyoHC-mRNA expression. Negative controls (medium controls, *n* = 3) did not show any PCR products.

In whole vessel segments, real-time RT-PCR analysis revealed mRNA expression of Kv2.1 channels (Figure 1A) along with eNOS transcripts (Ct for eNOS: 28.29 ± 0.32; *n* = 3). In contrast, mRNA for Kv2.2 channels was not detected in these samples, although more than 10 other specific primer pairs were screened, and Kv2.2 expression was detected in cDNA samples from the whole brain (Ct: 31.55 and 28.34; *n* = 2), indicating the functionality of the PCR assay. Amplification of polyA-RNA from total RNA by means of the TargetAmp™ 1-Round aRNA Amplification Kit 103 (Epicentre) or using a nested PCR approach also failed to detect Kv2.2-mRNA.

To ensure that Kv2.1 transcripts originate from smooth muscle cells and not from the endothelium, as indicated by the detection of eNOS transcripts in whole vessel segments, we performed ‘multiplex’ single-cell RT-PCR experiments on freshly dispersed smooth muscle cells. In these experiments, mRNA of Kv2.1 was detected in 10 out of a total of 11 cell samples (positive for MyoHC-mRNA) (Figure 1B); mRNA of Kv2.2 was not detected in the same samples (Figure 1B, at bottom). None of the negative controls (medium controls) shown in Figure 1B at the top yielded any PCR products. This mRNA-expression analysis thus revealed expression of Kv2.1 but not of Kv2.2 channels in smooth muscle cells of rat superior cerebellar arteries.

### 3.2. Kv2 Currents in Smooth Muscle Cells of Small Superior Cerebellar Arteries

**Figure 2:**
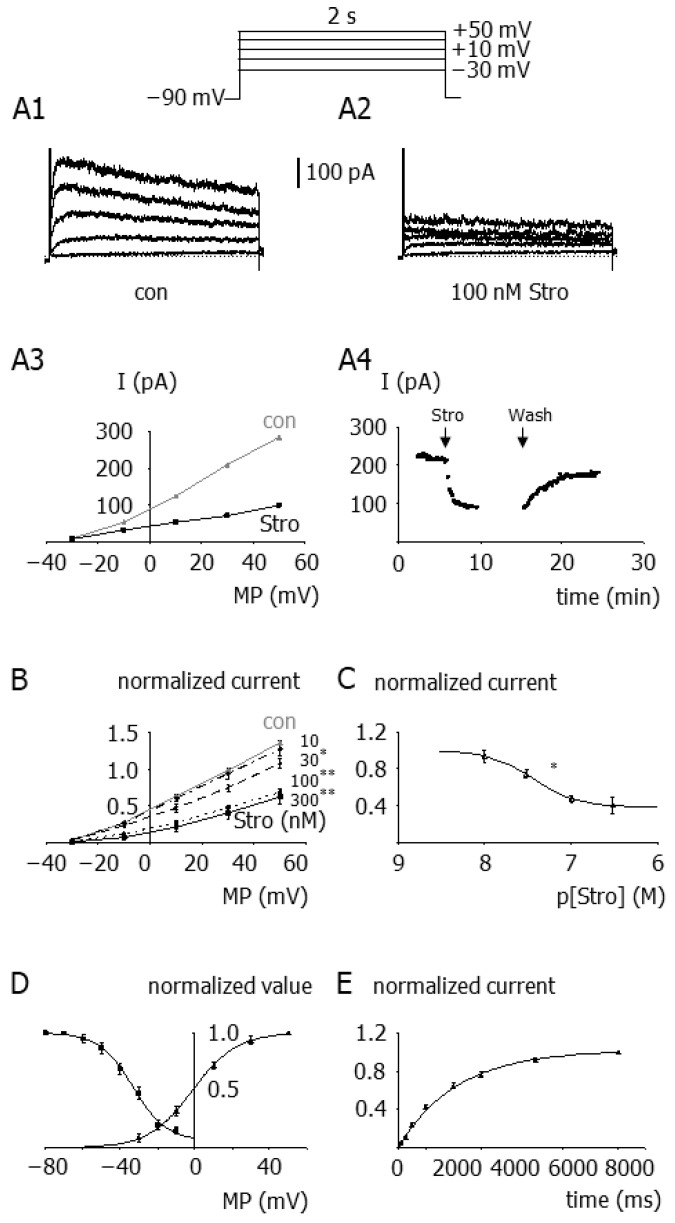
Kv2 currents in smooth muscle cells of small superior cerebellar arteries. (**A1**–**A4**) Example of the effect of stromatoxin on the Kv current. Example traces of the Kv current at different voltages in the absence (con) (**A1**) and presence of 100 nmol/L stromatoxin (Stro) (**A2**) and the current (I)-voltage (MP) relationship of these data (**A3**); the inset shows the voltage protocol. Time course of the effect of the addition (Stro) of 100 nmol/L stromatoxin and of its washout (Wash) on the Kv current at +30 mV (**A4**). The current-voltage relationship was obtained by applying 2 s voltage steps from a holding potential of −90 mV to test potentials between −30 and +50 mV in 20 mV increments. (**B**) Effect of stromatoxin at 10 nmol/L (*n* = 5), 30 nmol/L (*n* = 5), 100 nmol/L (*n* = 12), and 300 nmol/L (*n* = 6) on the current-voltage relationship of the Kv current. * *p* < 0.01; ** *p* < 0.001 (**C**) Concentration-response relationship of the effect of stromatoxin on the Kv current at +30 mV (*n* = 5–12). * *p* < 0.01 (**D**) Voltage-dependence of activation (**right curve**) and inactivation (**left curve**) of the stromatoxin-sensitive current. The stromatoxin-sensitive current was obtained by subtracting the current acquired in the presence of 100 nmol/L stromatoxin from the current observed before the addition of the inhibitor. The voltage dependence of activation was determined using the GHK-equation, data from the current-voltage relationships, and the Boltzmann equation. The inactivation was determined with voltage steps from holding potentials between −80 and +10 mV kept for 20 s to a test potential of +30 mV kept for 2 s. The stromatoxin-sensitive current exhibited voltage-dependence, with half-maximal activation at −1.3 ± 2.8 mV (*n* = 9) and a slope of 11.8 ± 1.0 mV (*n* = 9). Half-maximal inactivation occurred at −33.3 ± 1.9 mV (*n* = 10) with a slope of the curve of 8.9 ± 1.0 mV (*n* = 10). The steady state current at maximal inactivation was 6.4 ± 2.8% of the initial current (*n* = 10). (**E**) Recovery from inactivation of the stromatoxin-sensitive current. Following a holding potential of +30 mV kept for 10 s, the potential was set to −90 mV for 100, 250, 500, 1000, 2000, 3000, 5000, and 8000 ms and then stepped to a test potential of +30 mV. Recovery from inactivation occurred with a time constant of 2.12 ± 0.20 s (*n* = 10).

Patch-clamped smooth muscle cells displayed prominent Kv currents (Figure 2(A1)). The specific Kv2 channel inhibitor stromatoxin at 100 nmol/L [21,31] reduced the Kv current in these cells considerably (Figure 2(A2,A3)); this effect reached steady state after about 2 min and was reversible (Figure 2(A4)). The stromatoxin-induced inhibition of the Kv current was characterized by an EC_50_ of 36 nmol/L and a 61% reduction of the initial current at saturating inhibitor concentrations at +30 mV (Figure 2B,C). After subtraction of the stromatoxin-insensitive current determined using 100 nmol/L stromatoxin, the difference current exhibited prominent voltage-dependence of activation and inactivation (Figure 2D) and slow recovery from inactivation (Figure 2E). These pharmacological and biophysical properties resemble the characteristics of cloned Kv2.1 channels.

It should be noted that stromatoxin has been reported to potently inhibit Kv4.2 channels [31]. However, phrixotoxin, a specific inhibitor of Kv4.2 and Kv4.3 channels [37], did not affect the Kv current in the present study (normalized control current at +30 mV: 1.00 ± 0.02; current after addition of 3 µM phrixotoxin: 0.99 ± 0.03 (*n* = 5; *p* = 0.89)). The stromatoxin-insensitive current was blocked almost completely by 1 µmol/L DPO-1, a potent inhibitor of Kv1.5 channels [38], (normalized control current at +30 mV: 0.99 ± 0.01; current after addition of 300 nM stromatoxin + 1 µM DPO-1: 0.02 ± 0.01 (*n* = 4; *p* < 0.001)), indicating that this current is mediated mainly by Kv1 channels. In addition, stromatoxin has been shown not to inhibit BK channels in rat cerebral artery smooth muscle cells [11]. Together, our electrophysiological analysis shows that under the experimental conditions used, Kv2.1 channels carry a considerable part of the Kv-current in the smooth muscle cells studied.

### 3.3. Contribution of Smooth Muscle Kv2 Channels to the Myogenic Behavior of Small Superior Cerebellar Arteries

In intact vessels, vascular smooth muscle ion channels are exposed to a variety of regulatory factors, e.g., intracellular calcium, pH, protein kinases, and phosphatases. Unfortunately, the activity of these factors in intact vessels is not well-known enough to be able to mimic conditions found in intact vessels in patch-clamp studies on isolated cells. Therefore, a definitive understanding of the role of a particular ion channel can be obtained only in intact vessel preparations.

To exclude interference from Kv2.1 channels that may exist in the endothelium or in perivascular nerve endings, all experiments were performed after functional elimination of the endothelium and of sensory nerve endings (see also Section 2.2 in Material and Methods). Of note, both manipulations did not alter the myogenic properties of the vessels (Figure S1).

**Figure 3:**
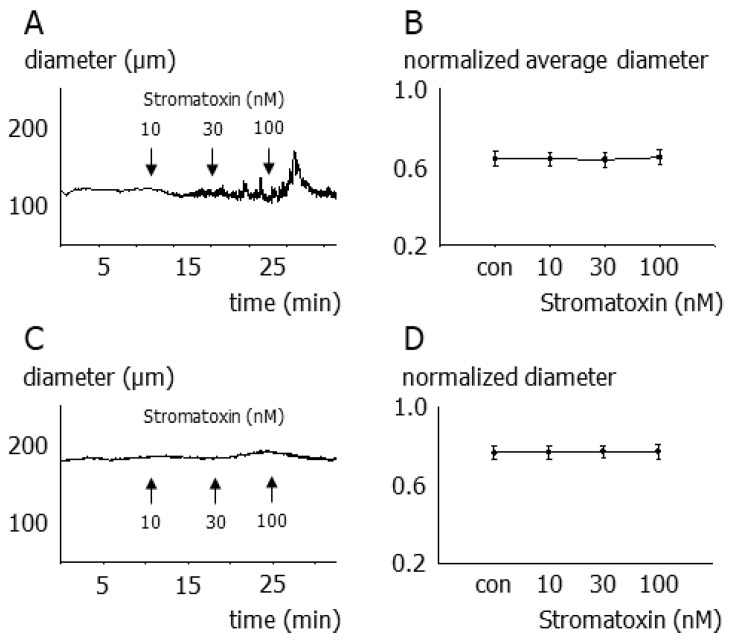
Contribution of smooth muscle Kv2 channels to the myogenic tone of small superior cerebellar arteries. (**A**) Example of the effect of stromatoxin on a vessel developing stromatoxin-induced vasomotion at 80 mmHg. (**B**) Concentration-response relationship of the effect of stromatoxin on average vessel diameter of vessels with vasomotion—transient diameter fluctuations were not taken into account (*n* = 8; *p* = 0.12). (**C**) Example of the effect of stromatoxin on a vessel not developing vasomotion at 80 mmHg. (**D**) Concentration-response relationship of the effect of stromatoxin on the diameter of vessels without vasomotion (*n* = 6; *p* = 0.58).

Application of stromatoxin to isobaric preparations of intact vessels induced vasomotion without affecting average vessel diameter in 38 out of 80 vessels studied. These effects are illustrated by the concentration-response relationship for stromatoxin shown in Figure 3A,B. Further, stromatoxin did not induce any diameter change in 42 out of 80 vessels studied, as exemplified by the concentration-response relationship shown in Figure 3C,D.

**Figure 4:**
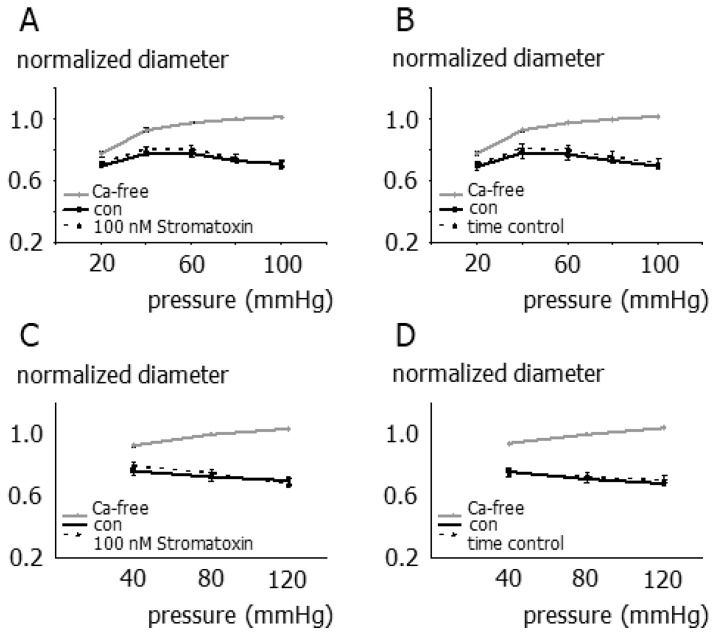
Contribution of smooth muscle Kv2 channels to the myogenic response of small superior cerebellar arteries not exhibiting vasomotion (**A**) Pressure-diameter relationship in the absence (con) and presence of stromatoxin (*n* = 7; *p* = 0.26). Stromatoxin was applied at 15 mmHg. (**B**) Two pressure-diameter relationships in the absence of stromatoxin were obtained with the same time response as in the experiments shown in A (*n* = 6; *p* = 0.13). (**C**) Pressure-diameter relationship in the absence (con) and presence of stromatoxin (*n* = 6; *p* = 0.25). Stromatoxin was applied at 80 mmHg. (**D**) Two pressure-diameter relationships in the absence of stromatoxin were obtained with the same time response as in the experiments shown in (**C**) (*n* = 6; *p* = 0.65).

In vessels not exhibiting vasomotion, two different protocols were used to study the effect of stromatoxin on myogenic reactivity. First, as in previous studies reporting a stromatoxin-induced increase in myogenic tone [24,25], myogenic reactivity was tested between 20 and 100 mmHg using 20 mmHg pressure increments; stromatoxin was applied at 15 mmHg. Second, myogenic reactivity was also tested between 40 and 120 mmHg using 40 mmHg pressure increments, where stromatoxin was applied at 80 mmHg prior to testing myogenic reactivity. Independent of the protocol used, stromatoxin did not affect myogenic reactivity (Figure 4A–D).

**Figure 5:**
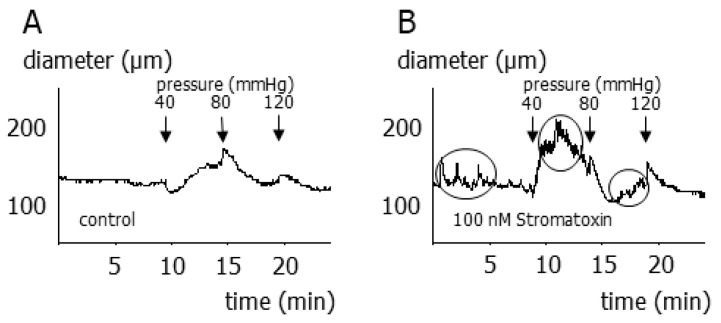
Contribution of smooth muscle Kv2 channels to the myogenic response of small superior cerebellar arteries exhibiting irregular vasomotion (**A**) Example of the myogenic response in the absence (control) of stromatoxin. (**B**) Example of the myogenic response in the presence of 100 nmol/L stromatoxin in a vessel showing random diameter oscillations (see encircled parts of the diameter recording) (*n* = 5).

In vessels exhibiting stromatoxin-induced vasomotion, two different patterns of vasomotion were observed. First, vasomotion occurred as sudden diameter fluctuations without any periodicity (9 out of 38 vessels) (for example, see the encircled parts of the diameter recording in Figure 5B). Under these conditions, pressure changes facilitated the occurrence of irregular vasomotion, disturbing the myogenic response completely (see Figure 5B compared to the control without stromatoxin in Figure 5A). This chaotic vasomotion made it impossible to identify a steady state in the diameter response after a pressure change. Thus, summarized data from these experiments cannot be presented.

**Figure 6:**
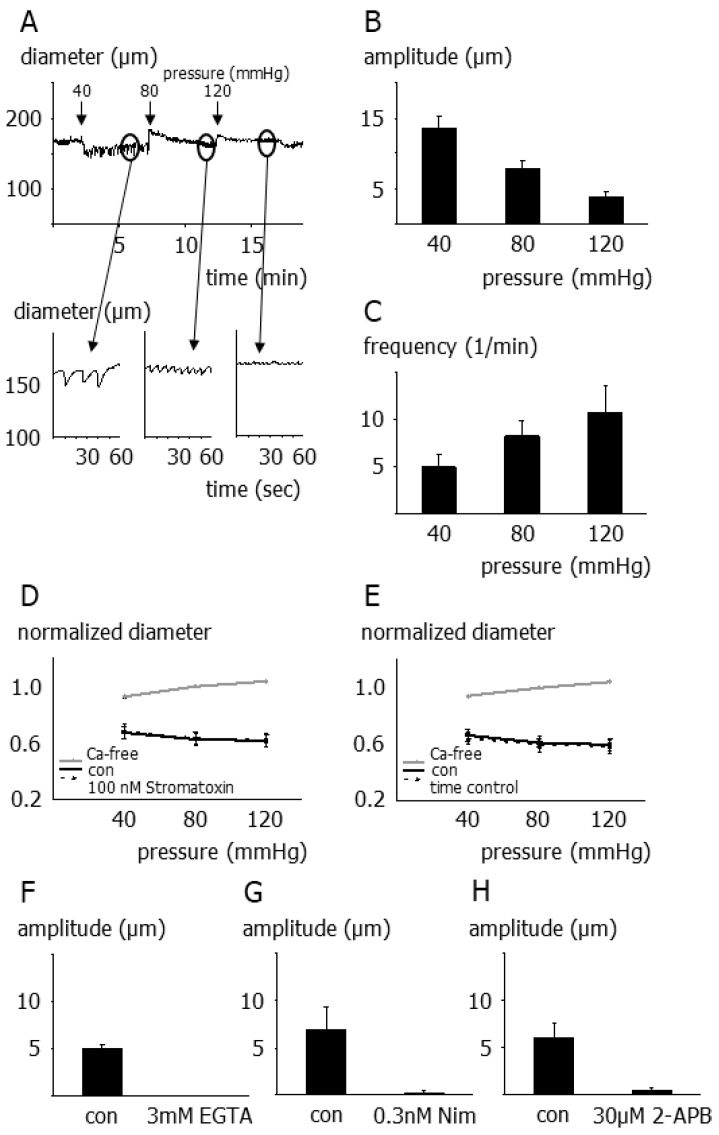
Contribution of smooth muscle Kv2 channels to the myogenic response of small superior cerebellar arteries exhibiting rhythmical vasomotion (**A**) Example of the myogenic response in the presence of 100 nmol/L stromatoxin in a vessel showing rhythmical diameter oscillations (see insets for more detail). (**B**) Effect of pressure on oscillation amplitude in the presence of 100 nmol/L stromatoxin (*n* = 5; *p* < 0.01). (**C**) Effect of pressure on oscillation frequency in the presence of 100 nmol/L stromatoxin (*n* = 5; *p* < 0.01). (**D**) Pressure-diameter relationship in the absence (con) and presence of stromatoxin (*n* = 7; *p* = 0.82). In the presence of stromatoxin, diameter is represented by its average value. (**E**) Two pressure-diameter relationships in the absence of stromatoxin were obtained with the same time response as in the experiments shown in € (*n* = 7; *p* = 0.17). (**F**) Effect of 3 mmol/L EGTA on oscillation amplitude in the presence of 100 nmol/L stromatoxin (*n* = 4; *p* < 0.001). (**G**) Effect of 0.2 nmol/L nimodipine (Nim) on oscillation amplitude in the presence of 100 nmol/L stromatoxin (*n* = 4; *p* < 0.05). (**H**) Effect of 10–30 µmol/L 2-APB on oscillation amplitude in the presence of 100 nmol/L stromatoxin (*n* = 4; *p* < 0.05).

Second, stromatoxin-induced vasomotion appeared as rhythmical vasomotion (29 out of 38 vessels) (for examples, see Figure 6A insets). In this case, pressure changes destabilized myogenic tone by modifying vasomotion characteristics considerably. Indeed, a rise in pressure decreased vasomotion amplitude (Figure 6B) and increased vasomotion frequency (Figure 6C). If vessel diameter was represented by its average value, stromatoxin did not affect the myogenic response (Figure 6D,E).

Of note, stromatoxin-induced vasomotion was abrogated by the addition of (i) 3 mmol/L EGTA, a chelator of extracellular calcium ions (Figure 6F), (ii) 0.3 nmol/L nimodipine, a blocker of voltage-dependent calcium channels (Figure 6G), and (iii) 10–30 µmol/L 2-APB, a blocker of capacitative and non-capacitative calcium entry pathways (Figure 6H).

## 4. Discussion

The principal findings of our study are that (i) Kv2.1 is the predominantly expressed Kv2 channel subtype in smooth muscle cells of rat superior cerebellar arteries, and (ii) this channel provides a novel type of negative feedback mechanism in myogenic autoregulation by preventing vasomotion. This novel action of the channel is important as it safeguards the myogenic response and could thus play a crucial role in controlling arterial tone and myogenic autoregulation in the cerebral circulation.

### 4.1. Kv2 Channel Expression in Smooth Muscle Cells of Small Superior Cerebellar Arteries

The expression pattern of Kv2 channels in the cerebral circulation has not been defined sufficiently. We therefore thoroughly determined Kv2 channel expression in smooth muscle cells of the superior cerebellar arteries by using whole-tissue and single-cell RT-PCR. We were able to detect Kv2.1-mRNA in whole vessel segments. In contrast, we did not detect the mRNA of the closely related Kv2.2 channel. Since whole vessel homogenates contain a sufficient amount of material originating from endothelial cells, as shown by the successful detection of eNOS transcripts, we further validated our finding by using the ‘multiplex’ single-cell RT-PCR approach [36,39] at the single smooth muscle cell level. These experiments revealed that, indeed, smooth muscle cells contain Kv2.1-mRNA. Similar to the results from whole vessel extracts, we were not able to detect Kv2.2 transcripts. Thus, Kv2.1 channels are the predominantly expressed Kv2 channel subtype in rat small superior cerebellar arteries. The finding of Kv2.1 channel expression is in line with a number of previous studies demonstrating the existence of Kv2.1 channels in rat small arteries of the systemic circulation (for more details see [11,24,27,29,40,41,42,43,44]). However, Kv2.2 channels have not been studied as extensively as Kv2.1 channels [11,27,29,42,44]. Moreover, studies investigating Kv2.2 expression showed both its presence in some vessels as well as its absence in other vessels [24,27,29,40,41,43]. In our study, we were not able to detect Kv2.2 channels, although we were able to detect Kv2.2 channel expression in brain extracts, which demonstrates the functionality of our RT-PCR assay. In conclusion, our study extends current knowledge about Kv2 channel expression by demonstrating Kv2.1 but not Kv2.2 channel expression in smooth muscle cells of rat small superior cerebellar arteries.

### 4.2. Kv2 Currents in Smooth Muscle Cells of Small Superior Cerebellar Arteries

Four main classes of potassium channels have been found in smooth muscle cells from small arteries: Kv channels, BK channels, ATP-sensitive potassium channels, and inward rectifying potassium channels [45]. In freshly isolated smooth muscle cells from rat superior cerebellar arteries, neither glibenclamide, an inhibitor of ATP-sensitive potassium channels, nor barium, an inhibitor of inward rectifying potassium channels, produced any effect on outward currents. We observed BK currents, which, however, were blocked by iberiotoxin, a specific inhibitor of BK channels [34], to unmask Kv-currents. Under these conditions, the outward current observed in this study showed characteristics—outward rectification, slow activation, and inactivation—typical for Kv currents found in smooth muscle cells from a number of other small artery preparations [11,27,29,41].

Importantly, most of the Kv current, namely 61%, was sensitive to stromatoxin, a high-affinity, selective inhibitor of Kv2 channels [21,31], suggesting that Kv2.1 channels carry a large portion of Kv currents in isolated smooth muscle cells of rat small superior cerebellar arteries. This is supported by our observation that the action of stromatoxin on the Kv current—relative fast block, slow but complete reversibility, the EC_50_, and voltage-dependence of block—resembles very closely the blocking effect of stromatoxin on heterologous expressed Kv2.1 channels [31]. Moreover, the characteristics of the stromatoxin-sensitive current observed in the present study are consistent with those for currents carried by Kv2.1 channels in other vascular smooth muscle cells [11,21,24,27,40,42] and by cloned Kv2.1 channels [17,24,31,41,46].

Moreover, based on the biophysical properties of native stromatoxin-sensitive currents and currents evoked by homologously expressed Kv2.1 and heterologously expressed Kv2.1/Kv9.3 channels, it was suggested that the native current in rat middle cerebral arteries [24] and rat mesenteric arteries [41] is carried by heteromultimeric Kv2.1/Kv9.3 channels. The properties of the stromatoxin-sensitive current observed in the present study are consistent with this suggestion.

Notably, a considerable window current of the Kv2.1 current was observed at physiological membrane potentials (see Figure 2D). The smaller stromatoxin-insensitive current was sensitive to DPO-1, a potent inhibitor of Kv1.5 channels [38]. This demonstrates a significant contribution of Kv1 channels to the total Kv current in small superior cerebellar artery smooth muscle cells, which is in line with previous findings from other cerebral arteries [15,19,22]. Importantly, stromatoxin does not affect cloned homomeric Kv1.1–1.6 and heteromeric Kv1.2/1.5 channels [11,31]. However, it should be noted that stromatoxin has been reported to potently inhibit Kv4.2 channels [31]. Yet, phrixotoxin, a specific inhibitor of Kv4.2 and Kv4.3 channels [37], did not affect the Kv current in the present study. In addition, stromatoxin has been shown to have no effect on vascular smooth muscle BK channels or expressed L-type calcium channels [11]. Finally, stomatoxin had no effect on Kv currents or vessel diameter in Kv2.1 channel knockout mice [21].

In conclusion, we present the first detailed biophysical analysis of stromatoxin-sensitive Kv currents in vascular smooth muscle cells and determine the concentration-dependence of the effect of stromatoxin on this current, extending the pioneering work by Amberg et al. 2006 [11] and Zhong et al. 2010 [24]. Thus, by using the most direct method to characterize plasmalemmal ion channels at the molecular level, the patch-clamp technique, we provide compelling evidence that the stromatoxin-sensitive Kv current in freshly isolated rat small superior cerebellar artery smooth muscle cells is an important part of the Kv current in these cells and is carried by Kv2.1 channels.

### 4.3. Contribution of Smooth Muscle Kv2 Channels to the Myogenic Behavior of Small Superior Cerebellar Arteries

Considering the large Kv2.1 current in isolated smooth muscle cells of this artery, we sought to define its contribution to myogenic tone and the myogenic response in intact vessels. Studies on intact vessels are crucial because experimental conditions (e.g., level of intracellular calcium and pH, activity of protein kinases and phosphatases) in the previously reported patch-clamp experiments are very different from intact vessel preparations under pressure. Thus, evidence for the existence of a certain current, here the Kv2.1 current, in isolated cells is by no means proof of the functional role of this current in intact vessel preparations.

In order to specifically address the role of smooth muscle cell Kv2.1 channels, most experiments were performed after functional elimination of the endothelium and of sensory nerve endings.

Surprisingly, stromatoxin produced unanticipated effects on isobaric preparations of intact small superior cerebellar arteries. Thus, stromatoxin application at constant pressure induced vasomotion without affecting average vessel diameter in 48% of the vessels under investigation, revealing a loss of control of vessel tone. Stromatoxin did not induce any diameter response, including vasomotion, in the other 52% of the vessels. The occurrence of vasomotion was observed in vessels with stronger and weaker myogenic tone. At the moment, we do not have experimental data explaining why only about half of the vessels tested produced vasomotion in response to stromatoxin. Preliminary findings obtained in the course of other projects (e.g., [20,47,48]) suggest that stromatoxin-induced vasomotion seems to depend on a delicate interplay with other potassium channels and possibly also depolarizing chloride and TRP channels. Blockade of some of the potassium channels alone, however, was not able to induce vasomotion. In contrast to these findings, previous studies reported that stromatoxin caused sustained vessel constriction at either 80 or 10 mmHg in rat middle cerebral arteries [11,24,25] and at 60 mmHg in mouse posterior cerebral arteries [32]. Because of its complexity, this issue has to be addressed in future studies.

Moreover, stromatoxin was reported to affect the myogenic response by enhancing myogenic tone without evoking vasomotion in the pioneering studies on the functional role of Kv2.1 channels in myogenic reactivity by Amberg et al. 2006 [11] and Zhong et al. 2010 [24,25]. Therefore, we proceeded by studying the effect of stromatoxin on the myogenic response of those small superior cerebellar arteries that did not develop vasomotion. To accomplish this, two different pressure protocols were employed: (i) the same protocol as in the previously published studies where stromatoxin was applied at low pressure [24,25] and (ii) a pressure protocol extending to larger pressures where stromatoxin was applied at higher pressure. In contrast to previous studies [24,25], albeit using the same method—isobaric myography—stromatoxin did not affect myogenic reactivity in our study, independent of the protocol used. Thus, the findings of the preset study suggest the new idea that in rat small superior cerebellar arteries, the contribution of Kv2 channels to myogenic reactivity differs from the well-known negative feedback mechanisms by which these channels and other Kv channels weaken myogenic autoregulation.

The most unexpected effect of stromatoxin observed in the present study was the induction of vasomotion, an effect never reported previously (see, in particular, Amberg et al. 2006 [11], Zhong et al. 2010 [24,25], and Nieves-Cintron et al. 2015 [32]). It should be noted, however, that in those studies, other cerebral arteries, the middle and posterior cerebral arteries, were used. Currently, the reason for the occurrence of varying effects of stromatoxin in different vessels is unclear. The clarification of this question requires a detailed comparative investigation that is, however, beyond the scope of the present study. Importantly, in vessels exhibiting stromatoxin-induced vasomotion in the present study, stromatoxin affected myogenic reactivity, albeit in an unusual way. First, if vasomotion occurred as sudden diameter fluctuations, pressure changes further facilitated the occurrence of either strong vasodilations or vasoconstrictions, thus disturbing the myogenic response completely. This finding shows that Kv2.1 channels protect the myogenic response by preventing chaotic vasomotion.

Second, if vasomotion was rhythmical, the myogenic response, based on average vessel diameter, was not altered, in contrast to the findings in the studies by Amberg et al. 2006 [11] and Zhong et al. 2010 [24,25]. The finding of the present study seems to indicate that Kv2.1 channels do not contribute to myogenic autoregulation in the presence of rhythmical vasomotion. However, this is a fallacy. It should be kept in mind that flow autoregulation is the ultimately essential process determining organ oxygen supply. In the absence of vasomotion, flow autoregulation is directly related to vessel diameter autoregulation. Since the latter can be measured directly, it is usually reported in studies on myogenic autoregulation. However, this direct relationship is not valid in the presence of vasomotion. In this case, one has to consider that an increase in vasomotion amplitude at an unaltered average vessel diameter increases flow [49]. Exactly this has been observed in our study: pressure increases in the presence of stromatoxin-induced (i) a change in average vessel diameter that was not different from that in the absence of the substance and (ii) a decrease in vasomotion amplitude. At high pressure, vasomotion amplitude was rather small, resulting in not much difference in flow in the presence and absence of stromatoxin. However, at low pressure, vasomotion amplitude was large. Thus, in the presence of stromatoxin, compared to its absence, flow is larger and closer to maximum flow, resulting in a smaller flow reserve, another functionally important factor besides flow itself controlled by myogenic autoregulation. Thus, the present study provides the novel finding that, in the presence of rhythmical vasomotion, Kv2.1 channels ensure the existence of a sufficient flow reserve in the lower physiological pressure range by preventing rhythmical vasomotion. Independent of vasomotion pattern, all findings together propose that Kv2.1 channels provide a novel type of negative feedback mechanism by which these channels safeguard myogenic autoregulation by preventing vasomotion.

The suggested role of Kv2.1 channels in myogenic autoregulation is consistent with current concepts explaining vasomotion as a process based on local calcium oscillations produced by calcium release and uptake by calcium stores in single smooth muscle cells that are synchronized in most vessels by ion channels in the cell membrane [50,51,52,53,54]. Being a relatively large peptide applied extracellularly, stromatoxin is not able to interfere with the intracellular calcium oscillator. In contrast, stromatoxin can easily reach the ion channels responsible for the synchronization of the calcium oscillator. The observation that Kv2.1 channels prevent vasomotion is most likely explained by an antagonizing action of the outward current carried by these channels on an inward current producing smooth muscle cell synchronization that is required for vasomotion [52,53]. This notion is supported experimentally by our observations that stromatoxin-induced vasomotion was abrogated by the addition of (i) EGTA, a chelator of extracellular calcium ions; (ii) nimodipine, a blocker of voltage-dependent calcium channels; and (iii) 2-APB, a blocker of capacitative and non-capacitative calcium entry pathways, interventions generally known to stop vasomotion [50].

Of note, recently, Kv2.1 channels have been suggested to play a dual role [21]: a canonical conductive and/or a structural role. Our patch-clamp data show that smooth muscle cells isolated from the studied vessel possess a considerable stromatoxin-sensitive outward current. This suggests that Kv2.1 channels can play a conductive role in these cells. In isolated arteries, stromatoxin induced vasomotion in a considerable number of vessels. This finding is consistent with the conductive role of Kv2.1 channels because ion channels have been shown to contribute to vasomotion [50]. In a number of vessels, stromatoxin was observed to have no effect on vessel diameter. Yet, this observation is not inconsistent with the conductive role of Kv2.1 channels. Of note, dominating potassium conductances can completely mask the functional role of smaller potassium conductances [48]. Future studies systematically exploring the interaction of different potassium channels during the induction of vasomotion should clarify this issue. Additionally, a structural role for Kv2.1 channels has been suggested [21], whereby Kv2.1 channels form clusters, especially with calcium channels. This was demonstrated to lead to a more depolarized membrane potential and a stronger myogenic tone. Interestingly, in our experiments, stromatoxin-induced vasomotion seemed to be more likely to occur in vessels exhibiting stronger tone. These data may indicate that Kv2.1 channels may have a larger structural role in vessels exhibiting vasomotion compared to vessels not exhibiting vasomotion. If this is the case, the observation that the structural role of Kv2.1 channels is larger in vessels from female animals compared to male animals may indicate that vasomotion may occur more frequently in vessels from female animals. All these suggestions require extensive experimentation and should be addressed in future studies.

Under functional aspects, vasomotion is not something that should be avoided under all circumstances. Although the physiological significance of vasomotion is yet to be clearly defined, it has been suggested that for the same average diameter, vasomotion ensures increased flow conductance, effective dialysis of tissues, and vasomotive oxygen delivery [50,51,52,53]. Indeed, vasomotion exists in many vessels, mainly arterioles [50,51,52], which, incidentally, do not contribute much to myogenic autoregulation [2,3,4,5]. Thus, the presence of Kv2.1 channels in small cerebral arteries together with their absence in parenchymal arterioles [54] seems to be an important factor limiting the occurrence of vasomotion in arterioles. In conclusion, by preventing vasomotion in small arteries, Kv2.1 channels are not only safeguarding myogenic autoregulation but are also contributing to the regional allocation of myogenic autoregulation to small arteries and vasomotive regulation to arterioles.

Previous studies on the mechanisms of myogenic autoregulation had discovered a number of pathways mediating pressure-induced contraction and a few pathways responsible for negative feedback dilation limiting myogenic vasoconstriction. Our study suggests another negative feedback mechanism contributing to myogenic autoregulation that prevents vasomotion and identifies the molecular key player of this mechanism, the Kv2.1 channel. Thus, our study contributes to a better understanding of the mechanisms of myogenic autoregulation by suggesting (i) that the previously reported important functional role of Kv2.1 channels, the limitation of myogenic constriction, cannot be translated to other vessels without experimental testing and (ii) that the functional role of Kv2.1 channels in myogenic autoregulation may also be to ensure the existence of a sufficient flow reserve in the lower physiological pressure range by preventing rhythmical vasomotion.

## 5. Conclusions

In summary, the data of the present study provide the first evidence that smooth muscle Kv2.1 channels contribute to myogenic behavior in a previously unrecognized fashion, providing a novel type of negative feedback mechanism in myogenic autoregulation by primarily preventing vasomotion. Vasomotion, if unopposed, has the capability to destabilize the myogenic tone and disturb the myogenic response. We therefore suggest a substantially extended concept that smooth muscle Kv2.1 channels are safeguards of myogenic autoregulation and stable organ perfusion and could have protective effects in cardiovascular disease states.

## Data Availability

The datasets generated during and/or analyzed during the current study are available from the corresponding author on reasonable request.

## Supplementary Materials

The following supporting information can be downloaded at: https://www.mdpi.com/article/10.3390/cells12151989/s1, Figure S1: The myogenic response of small superior cerebellar arteries.
