# Supplementary material for: Kv2.1 Channels Prevent Vasomotion and Safeguard Myogenic Reactivity in Rat Small Superior Cerebellar Arteries
Source: Cells. 2023 Aug 2;12(15):1989. doi: 10.3390/cells12151989 (PMC10416909; doi:10.3390/cells12151989)
Supplement: Supplementary file 1 [file cells-12-01989-s001.zip › cells-2518942-supplementary.pdf]

Article

Kv2.1 Channels Prevent Vasomotion and Safeguard Myogenic Reactivity in Rat Small Superior Cerebellar Arteries

Kristina Här [1,†], Natalia N. Lysenko [1,2,†], Daniela Dimitrova [3], Torsten Schlüter [4], Olga Zavaritskaya [1], Andrej G. Kamkin [2], Mitko Mladenov [2,5], Olaf Grisk [6], Ralf Köhler [7], Hristo Gagov [8] and Rudolf Schubert [1,9,*]

[1] European Center of Angioscience (ECAS), Research Division Cardiovascular Physiology, Medical Faculty Mannheim, Heidelberg University, 68167 Mannheim, Germany
[2] Department of Physiology, N. I. Pirogov Russian National Research Medical University, 117997 Moscow, Russia
[3] Institute of Biophysics and Biomedical Engineering, Bulgarian Academy of Sciences, 1113 Sofia, Bulgaria
[4] Institute of Physiology, Universitätsmedizin Greifswald, 17475 Greifswald, Germany
[5] Institute of Biology, Faculty of Natural Sciences and Mathematics, University of Ss. Cyril and Methodius, 1000 Skopje, North Macedonia
[6] Institute of Physiology, Brandenburg Medical School Theodor Fontane, 16816 Neuruppin, Germany
[7] ARAID-IACS, UIT University Hospital Miguel Servet, 50009 Zaragoza, Spain
[8] Department of Animal and Human Physiology, Faculty of Biology, Sofia University 'St. Kliment Ohridski', 1164 Sofia, Bulgaria
[9] Physiology, Institute of Theoretical Medicine, Faculty of Medicine, University of Augsburg, Universitätsstrasse 2, 86159 Augsburg, Germany
* Correspondence: rudolf.schubert@med.uni-augsburg.de
† These authors contributed equally to this work.

Supplementary

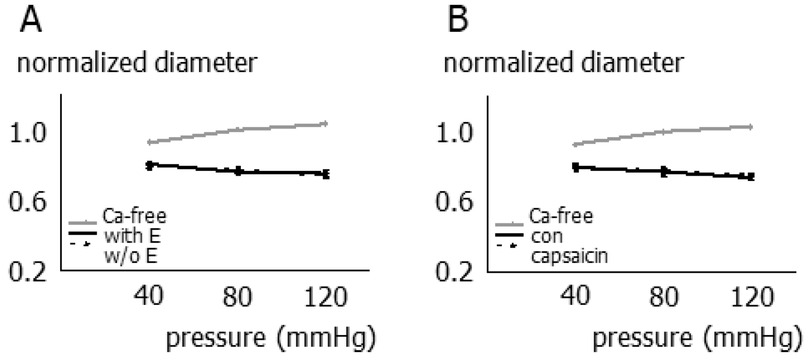

Figure S1. The *myogenic response of small superior cerebellar arteries*. (**A**) Pressure-diameter relationship of endothelium-intact (with E) and endothelium-denuded (w/o E) vessels (n = 6; p = 0.86). For comparison the passive vessel response in the absence of extracellular calcium (Ca-free) is shown. (**B**) Pressure-diameter relationship of capsaicin-treated (capsaicin; 10 µmol/L) and untreated (con) vessels (n = 6; p = 0.61).
